# O.4.6-7 The role of external providers for Physical Education and extracurricular physical activity in Irish primary schools

**DOI:** 10.1093/eurpub/ckad133.226

**Published:** 2023-09-11

**Authors:** Mairéad Teehan, Lisa Kelly, Aoife Lane, Niamh Ní Cheilleachair, Kieran Dowd

**Affiliations:** Technological University of the Shannon, Midlands-Midwest, Athlone Campus, Ireland; m.teehan@research.ait.ie; Technological University of the Shannon, Midlands-Midwest, Athlone Campus, Ireland; m.teehan@research.ait.ie; Technological University of the Shannon, Midlands-Midwest, Athlone Campus, Ireland; m.teehan@research.ait.ie; Technological University of the Shannon, Midlands-Midwest, Athlone Campus, Ireland; m.teehan@research.ait.ie; Technological University of the Shannon, Midlands-Midwest, Athlone Campus, Ireland; m.teehan@research.ait.ie

## Abstract

**Purpose:**

In Irish primary schools, Physical Education (PE) is taught by generalist teachers. While PE is critical in developing essential skills for lifelong physical activity (PA) participation, studies show teacher confidence and competence to teach PE is low due to negative personal experiences and insufficient training. Many schools utilise external providers to supplement PE provision and provide extracurricular PA. However, this provision is largely unregulated, without clear distinction between the two. This study aims to examine the role of external providers for PE and PA in Irish primary schools.

**Methods:**

An online survey for principals and teachers was developed and validated using a modified Delphi technique. It included 57 questions examining current practices for PE provision schools. A link to the survey was emailed to all Irish primary schools and shared on social media. SPSS version 27 was used for data analysis.

**Results:**

Of the 513 respondents, 81.7% were female, 17.9% male and 0.4% didn’t say. A majority (82%) regularly use external providers for extracurricular PA, which takes place mostly during the school day (89%). Although intended as activity not related to PE provision, it is used by teachers as the only PE provided either sometimes (45.5%) or all of the time (21.6%). External providers teach specific strands of the PE curriculum in 52.9% of schools, with Games being the strand most provided. While this provision is taking place, most teachers stay with their class and assist (61.9%).

**Conclusion:**

Results show external providers are extensively used in Irish primary schools to supplement PE provision rather than solely providing extracurricular PA. Games being the most commonly delivered strand by external providers is reflective of the number of sports coaches providing this service to schools. Teachers using extracurricular PA as their PE can be problematic as external providers often lack suitable qualifications and provision may not align with PE curriculum guidelines. Support is needed for schools to provide good quality PE and approaches such as regulating the use of external providers or engaging specialist PE teachers to support delivery should be examined in order to improve PE in Irish primary schools.

